# Isolation and Characterization of Pepper Genes Interacting with the CMV-P1 Helicase Domain

**DOI:** 10.1371/journal.pone.0146320

**Published:** 2016-01-11

**Authors:** Yoomi Choi, Min-Young Kang, Joung-Ho Lee, Won-Hee Kang, JeeNa Hwang, Jin-Kyung Kwon, Byoung-Cheorl Kang

**Affiliations:** Department of Plant Science, Plant Genomics & Breeding Institute, and Research Institute of Agriculture and Life Sciences, Seoul National University, Seoul, 151–921, Korea; National University of Singapore, SINGAPORE

## Abstract

*Cucumber mosaic virus* (CMV) is a destructive pathogen affecting *Capsicum annuum* (pepper) production. The pepper *Cmr1* gene confers resistance to most CMV strains, but is overcome by CMV-P1 in a process dependent on the CMV-P1 RNA1 helicase domain (P1 helicase). Here, to identify host factors involved in CMV-P1 infection in pepper, a yeast two-hybrid library derived from a *C*. *annuum* ‘Bukang’ cDNA library was screened, producing a total of 76 potential clones interacting with the P1 helicase. Beta-galactosidase filter lift assay, PCR screening, and sequencing analysis narrowed the candidates to 10 genes putatively involved in virus infection. The candidate host genes were silenced in *Nicotiana benthamiana* plants that were then inoculated with CMV-P1 tagged with the green fluorescent protein (GFP). Plants silenced for seven of the genes showed development comparable to *N*. *benthamiana* wild type, whereas plants silenced for the other three genes showed developmental defects including stunting and severe distortion. Silencing *formate dehydrogenase* and *calreticulin-3 precursor* led to reduced virus accumulation. *Formate dehydrogenase*-silenced plants showed local infection in inoculated leaves, but not in upper (systemic) leaves. In the *calreticulin-3 precursor*-silenced plants, infection was not observed in either the inoculated or the upper leaves. Our results demonstrate that *formate dehydrogenase* and *calreticulin-3 precursor* are required for CMV-P1 infection.

## Introduction

All viruses are dependent on their host factors for a successful infection of hosts through the virus-host interactions. Physical interactions between viral components and host factors are required for replication, cell-to-cell movement, and systemic movement in viral pathogenesis [1]. Accordingly, host factors are essential components in most steps of virus infection [2, 3]. The outcome of such interactions determines host specificity and tissue specificity of virus strains [4, 5]. Without the interactions with host factors, viruses are unable to infect; hence, virus-host interactions can be utilized for development of virus-resistant crops [4].

*Cucumber mosaic virus* (CMV) has a very wide host range and is one of the most destructive viruses facing pepper production. CMV belongs to the *cucumovirus* group of the *Bromoviridae* family. CMV encodes five proteins from its tripartite genome [6–8]. CMV RNA1 encodes the CMV 1a protein, which is composed of the methyltransferase domain in its N-terminal part and the helicase (Hel) domain in the C-terminal part [9]. The 1a protein is involved in CMV replication and systemic infection [7]. CMV RNA2 encodes the CMV 2a and 2b proteins. The 2a protein has RNA polymerase activity, and the 2b protein affects the host range and acts as a suppressor of post-transcriptional gene silencing [10, 11]. CMV RNA3 encodes the movement protein (MP) and coat protein (CP) [12, 13], both of which are related to cell-to-cell movement of CMV [14].

A number of host factors related to infection have been identified, and the effects of host factor mutations on virus infection have been characterized in various plant species [15]. For example, *Nicotiana tabacum* thaumatin-like protein 1 (NtTLP1), which directly interacts with CMV 1a protein, plays an important role in CMV replication and/or movement in *Nicotiana benthamiana* [9]. *Tobacco* CMV 1a interacting protein 1 (Tcoi1) directly interacts with the CMV 1a methyltransferase (MT) domain, and overexpression of *Tcoi1* enhances CMV infection while silencing of *Tcoi1* decreases virus infection [16]. Tsi1-interacting protein 1 (Tsip1) strongly interacts with CMV 1a and CMV 2a protein which has the GDD motif typical for RNA-dependent RNA polymerase (RdRp), and forms the viral replicase complex. This replicase complex controls CMV multiplication in tobacco plants [17]. Additionally, knockout of the *Suppressor of SA insensitive 2* (*SSI2)* gene, which encodes a plastid-localized stearoyl-ACP desaturase, enhances resistance to CMV in *Arabidopsis*, and suppress viral multiplication and systemic movement [18].

*Capsicum annuum* ‘Bukang’, which contains a single dominant resistance gene *Cucumber mosaic resistance 1* (*Cmr1*), is resistant to CMV isolate-P0 strains (CMV-P0; CMV-Kor and CMV-Fny) [19]. However, a new isolate of CMV, to CMV isolate-P1 (CMV-P1), breaks *Cmr1*-mediated resistance [20]. Recently, it was reported that the Hel domain is a virulence factor for CMV-P1. The C-terminal region of the Hel domain is responsible for systemic infection by controlling viral replication and cell-to-cell movement [21].

Here, we screened for host genes that interact with the CMV-P1 Hel domain using a yeast two-hybrid system and studied their effects on CMV-P1 infection in *N*. *benthamiana*. We found that Formate dehydrogenase (FDH) and Calreticulin-3 precursor (CRT3) directly interact with the CMV-P1 helicase domain. In addition, CMV-P1 harboring the green fluorescent protein (GFP) was not detected in *FDH*- or *CRT3*-silenced *N*. *benthamiana* plants. Through enzyme-linked immunosorbent assay (ELISA), we demonstrated that the accumulation of CMV-P1 was significantly decreased in the silenced plants. Taking these results together, we suggest that host *FDH* and *CRT3* are required for the successful infection by CMV-P1.

## Materials and Methods

### Yeast Two-Hybrid Screening

Yeast transformation and analyses were performed using pBD-GAL4 Cam and pAD-GAL4-2 vectors (Agilent Technologies, Santa Clara, CA, USA). The CMV-P1 RNA1 helicase domain was amplified from a CMV-P1 cDNA clone provided by Professor Kook-Hyung Kim (Seoul National University, Korea) using PCR and cloned into pBD-GAL4 Cam vector. The resulting bait-containing pBD-GAL4 Cam vector was transformed into *Saccharomyces cerevisiae* strain YRG-2 and selected on synthetic complete medium (SC) lacking tryptophan (-Try) for 4 d at 30°C. For prey, a *C*. *annuum* ‘Bukang’ cDNA library was provided by Professor Doil Choi (Seoul National University, Korea). The prey vectors were transformed into the YRG-2 yeast strain containing the bait vector. Yeast co-transformants were incubated in the selection medium lacking tryptophan and leucine (SC-Try, Leu) for 5 d at 30°C. After co-transformation, each colony was streaked on synthetic complete medium (SC) lacking tryptophan, leucine and histidine (SC-Try, Leu, His) and grown for 5 d at 30°C. The pLAM5'-1/pAS2-1 and pTD1-1/pACT2 plasmids (Clontech, Mountain View, CA, USA) were used as a negative control, and pVA3-1/pAS2-1 and pTD1-1/pACT2 were used as a positive control.

### β-Galactosidase Filter Lift Assay

To identify interaction between candidate cDNAs and the CMV-P1 RNA1 helicase domain, co-transformed colonies were incubated in the selection synthetic complete liquid medium lacking tryptophan and leucine (SC-Try, Leu) for 3 d at 30°C. After 3 d, cells from each culture were incubated for 4 d at 30°C on synthetic complete medium lacking tryptophan and leucine (SC-Try, Leu) until the diameter of each colony was 0.4–0.7 mm. A 3MM filter (Whatman, Maidstone, Kent, UK) was placed in contact with all of the clones. The filter was then dipped in liquid nitrogen for 15 s and thawed for 1 min at room temperature. After three repeats of this step, the 3MM filter was soaked in Z buffer (60 mM Na_2_HPO_4_, 40 mM NaH_2_PO_4_⋅H_2_O, 10 mM KCl, 1 mM MgSO_4_⋅7H_2_O, 39 mM 2-mercaptoethanol, pH 7.0) with X-gal. The filter was then incubated for 8 h at 30°C in the dark and the signal was captured by a digital camera.

### PCR Screening

For PCR screening, primers were designed based on the pAD-GAL4-2.1 vector multiple cloning site (MCS) (S1 Table). Using these primers, the DNA fragments were amplified from the yeast clones containing candidate cDNAs by colony PCR. The PCR products were eluted and then cloned using the T-Blunt PCR cloning system (Solgent, Daejeon, South Korea). The ligated DNA fragments were transformed into *E*. *coli* strain DH10B and incubated in LB medium containing 50 mg/L kanamycin for selection. To confirm the cloning of PCR fragments, colony PCR was performed using AD vector-specific primers. The clones containing cDNA fragments were incubated in liquid LB medium containing 50 mg/L kanamycin for 1 d at 37°C in a shaking incubator. Plasmids were isolated from cultured cells using the AccuPrep^®^ Plasmid Mini Extraction Kit (Bioneer, Daejeon, South Korea) and sequences were determined (NICEM, Seoul National University, Seoul, South Korea).

### Sequence Analysis of Candidate Genes

The sequences of candidate genes were determined at the National Center for Biotechnology Information (NCBI, http://www.ncbi.nlm.nih.gov/) and blasted against *C*. *annuum* database (http://peppergenome.snu.ac.kr). The candidate gene name, description, and sequence ID are listed in Table 1 and S2 Table.

**Table 1:** List of candidates interacting with the CMV-P1 helicase domain.

Candidate gene	Putative Function	Sequence ID in *C*. *annuum*	Reference
*PPM*	Phosphomannomutase	CA08g10130	[26, 27]
*FDH*	Formate dehydrogenase	CA02g29530	[28, 29]
*CRT3*	Calreticulin-3 precursor	CA00g87370	[30, 31]
*UBI11*	Polyubiquitin 6PU11	CA00g79660	[32]
*Cysk*	Cysteine synthase	CA08g04930	[33, 34]
*AGP-S2*	ADP-glucose pyrophosphorylase large subunit	CA07g05920	[35]
*ARF1*	ADP-ribosylation factor 1	CA08g00830	[36]
*ARF*	ADP-ribosylation factor	CA01g22680	[36]
*H3*	Histone-H3	CA04g15130	[37–39]
*ARD*	Acireductone dioxygenase	CA03g06820	[40]

### Plasmid Construct for Virus-Induced Gene Silencing (VIGS)

The candidate genes were amplified from the *C*. *annuum* ‘Bukang’ cDNA using gene-specific primers designed based on the *C*. *annuum* database (http://peppergenome.snu.ac.kr) (S1 Table). A modified ligation-independent cloning system was used for cloning of the inserts into the TRV VIGS vector [22]. All PCR products were purified with the DNA Clean & Concentrator^™^-100 (Zymo Research, Irvine, CA, USA). The purified PCR products (15 fmol) were mixed with 5 mM dATP and treated with T4 DNA polymerase (Novagen, Darmstadt, Germany) at 22°C for 30 min. The TRV2-LIC vector was digested with *Pst1* and treated with T4 DNA polymerase with dTTP. The treated PCR product and TRV2-LIC vector were mixed in a 5:1 ratio and incubated at 65°C for 2 min and then transferred to 22°C for 10 min. A sample of the mixture (3 μL) was transformed into *E*. *coli* DH10B and transformed colonies were selected by colony PCR using LIC primers (S1 Table). Plasmids were extracted from identified colonies (Zymo Research, Irvine, CA, USA). Sequencing analysis was performed at the National Instrumentation Center for Environmental Management (Seoul National University, Seoul, Korea).

### Plant Materials and *Agrobacterium* Infiltration

*N*. *benthamiana* plants were grown for 4 weeks at 23°C with a 16-h light/8-h dark cycle. For the VIGS, the TRV VIGS system was used [23, 24]. TRV1 or TRV2 derivatives were transformed into *Agrobacterium* and the resulting strains were incubated in liquid LB medium containing antibiotics (50 mg/L kanamycin and 50 mg/L rifampicin) for 20 h at 30°C. The *Agrobacterium* cells were harvested and resuspended in infiltration medium (10 mM MgCl_2_, 10mM MES, 200μM acetosyringone), adjusted to 0.4 OD_600_, and incubated at room temperature with shaking for 4 h. *Agrobacterium* culture containing TRV1 was adjusted to 0.3 OD_600_ and incubated as described above. TRV1 and TRV2 or its derivatives were mixed in a 1:1 ratio and infiltrated into *N*. *benthamiana* at the four-leaf stage using a 1-mL syringe needle. At 12 d post infiltration (dpi), the silenced plants were used for further experiments.

### RNA Extraction and RT-PCR Analysis

Total RNA was extracted from leaves of *C*. *annuum* ‘Bukang’ and silenced *N*. *benthamiana* plants using GeneAll^R^Hybrid-R^™^ (Gene All Biotechnology, Seoul, South Korea) according to the manufacturer’s protocol. First-strand cDNA was synthesized from 4 μg total RNA using M-MLV reverse transcriptase (Promega, Madison, WI, USA) and oligo-(d)T primers (Bioneer, Daejeon, South Korea) according to the manufacturer’s protocol. For VIGS, the expression of candidate genes was analyzed by semi-quantitative RT-PCR and real-time PCR using gene-specific primers (S1 Table). For the semi-quantitative RT-PCR, *Actin* transcript was used as an endogenous control [25]. The real-time PCR was performed using a Lightcycler^®^480 instrument (Roche, Switzerland). Thermal cycling was as follows: denaturing at 95°C for 5 min, followed by 45 cycles of denaturing for 10 s, annealing at 60°C for 20 s and extension at 72°C for 15 s.

### Virus Inoculation and Evaluation of Resistance

CMV-P1-GFP inoculum was propagated in *N*. *benthamiana* at 23°C with a 16-h light/8-h dark cycle. The silenced plants were inoculated at the four-to-six-leaf stage, and the two oldest leaves were used for carborundum rub-inoculation with virus produced using grinding systemically infected leaves of *N*. *benthamiana* in 100 mM potassium phosphate buffer, pH 7.0 (1g tissue; 10 mL buffer). Plants were kept in a growth chamber at 25°C until symptom observation. Non- and mock-inoculated controls were included.

For *Agrobacterium*-mediated inoculation of CMV-P1-GFP, *Agrobacterium* carrying CMV-P1-GFP was incubated in liquid LB medium containing antibiotics (50 mg/L kanamycin and 50 mg/L rifampicin) for 20 h at 30°C. The *Agrobacterium* cells were harvested and resuspended in infiltration medium (10 mM MgCl_2_, 10 mM MES, 200 μM acetosyringone), adjusted to 0.4 OD_600_, incubated at room temperature with shaking for 3 h and then infiltrated into *N*. *benthamiana* at the four-leaf stage using a 1-mL syringe needle. Virus accumulation was tested using inoculated and upper non-inoculated leaves at 5 and 10 dpi by DAS-ELISA according to the manufacturer’s instructions (Agdia, Inc., Elkhart, USA). GFP was visualized using a confocal laser-scanning microscope (LSM 510; Carl Zeiss, Jena, Germany).

## Results

### Isolation of Candidate Genes Interacting with CMV Helicase Domain

To identify host genes interacting with the P1 Hel domain of CMV-P1, we performed yeast two-hybrid screening analysis. P1 Hel was cloned into a bait vector and a total 100,800 of *C*. *annuum* ‘Bukang’ cDNAs were cloned into prey vector. P1 Hel was co-transformed into YRG-2 yeast strain containing ‘Bukang’ cDNA (Table 2), and co-transformed yeast cells were grown on the non-selective synthetic complete medium (SC-Leu-Trp) and on the selective medium (SC-Leu-Try-His). When the 156 candidate interacting clones were subjected to a β-galactosidase filter lift assay to confirm interaction, only 82 showed a positive response. These 82 clones interacting with the P1 Hel domain were used for further study (Table 2).

**Table 2:** Summary of screen for *C*. *annuum* host proteins interacting with the CMV-P1 helicase domain.

Total number of clones	Number of selected clones			
	Y2H screening	β-galactosidase filter lift assay	PCR screening	Sequence analysis
100,080	156	82	80	78

### Sequence Analysis of Candidate Genes

**Fig 1:**
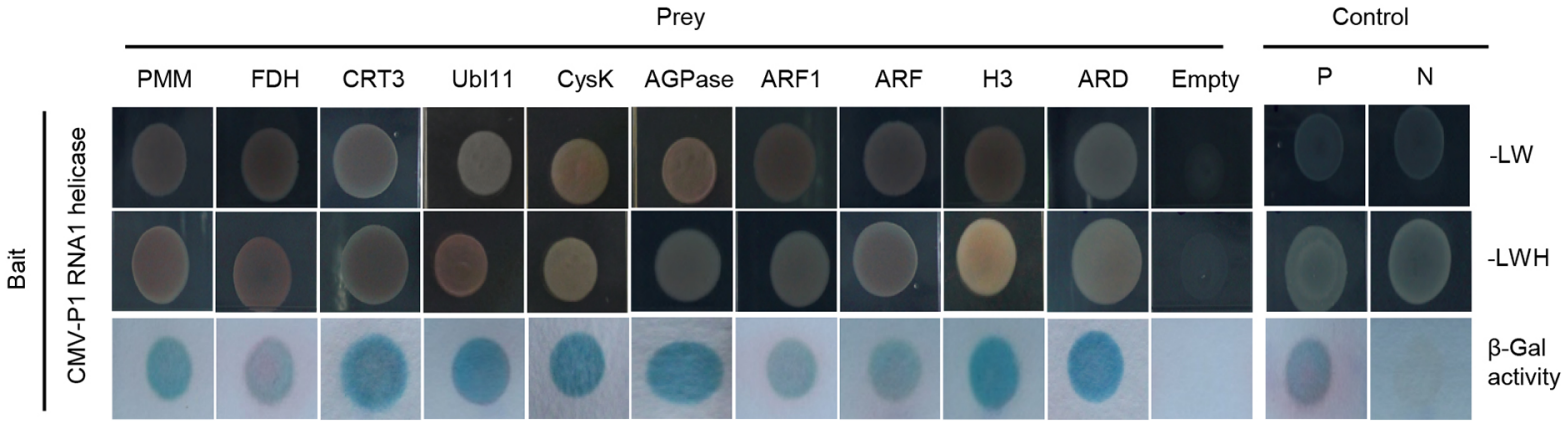
Interactions of candidate clones and the CMV-P1 RNA1 helicase domain using in the yeast two-hybrid. The bait was the CMV-P1 RNA1 helicase domain. For prey, PMM, Phosphomannomutase; FDH, Formate dehydrogenase; CRT3, Calreticulin-3 precursor; UBI11, Polyubiquitin 6PU11; CysK, Cysteine synthase; AGPase, ADP-glucose pyrophosphorylase; ARF1, ADP-ribosylation factor 1; ARF, ADP-ribosylation factor; H3, Histone-H3; ARD, Acireductone dioxygenase were used. P and N represent positive and negative controls, respectively. Empty is a bait vector alone. SD medium (-LW; lacking tryptophan and leucine and -LWH; lacking tryptophan, leucine, and histidine) was used to select for co-transformation. β-galactosidase (β-Gal) activity assays were performed according to the manufacturer’s protocol.

To investigate the sequences of the 82 candidate genes, we performed PCR from co-transformed colonies using a MCS primer for the pAD-GAL4-2.1 vector (S1 Table). Among the 82 clones tested, PCR products were successfully obtained from 78 clones (Table 2). The insert size varied from 401 bp up to 1498 bp. After sequencing PCR products, the putative function of genes were identified and genes were classified by according to their function (S2 Table). Among these genes, we selected 10 genes that were related to viral pathogens based on their NCBI database annotations and previous research. These included *PPM* (*phosphomannomutase*) [26, 27], *FDH* (*formate dehydrogenase*) [28, 29], *CRT3* (*calreticulin-3 precursor*) [30, 31], *UBI11* (*polyubiquitin 6PU11*) [32], *Cysk* (*cysteine synthase*) [33, 34], *AGPase* (*ADP-glucose pyrophosphorylase*) [35], *ARF1* (*ADP-ribosylation factor 1*) [36], *ARF* (*ADP-glucose pyrophosphorylase*) [36], *H3* (*histone-H3*) [37–39], and *ARD* (*acireductone dioxygenase*) [40] (Table 1 and Fig 1). To obtain full length sequences and check copy numbers of the genes in the pepper genome, the sequences were BLAST searched against the pepper genome database (http://peppergenome.snu.ac.kr). Most candidate genes were found to be single copy genes in pepper, except for *FDH* (S2 Table). Taking these results together, these 10 genes were selected as candidate host factors of CMV-P1.

### Silencing of the Candidate Genes in *N*. *benthamiana*

**Fig 2:**
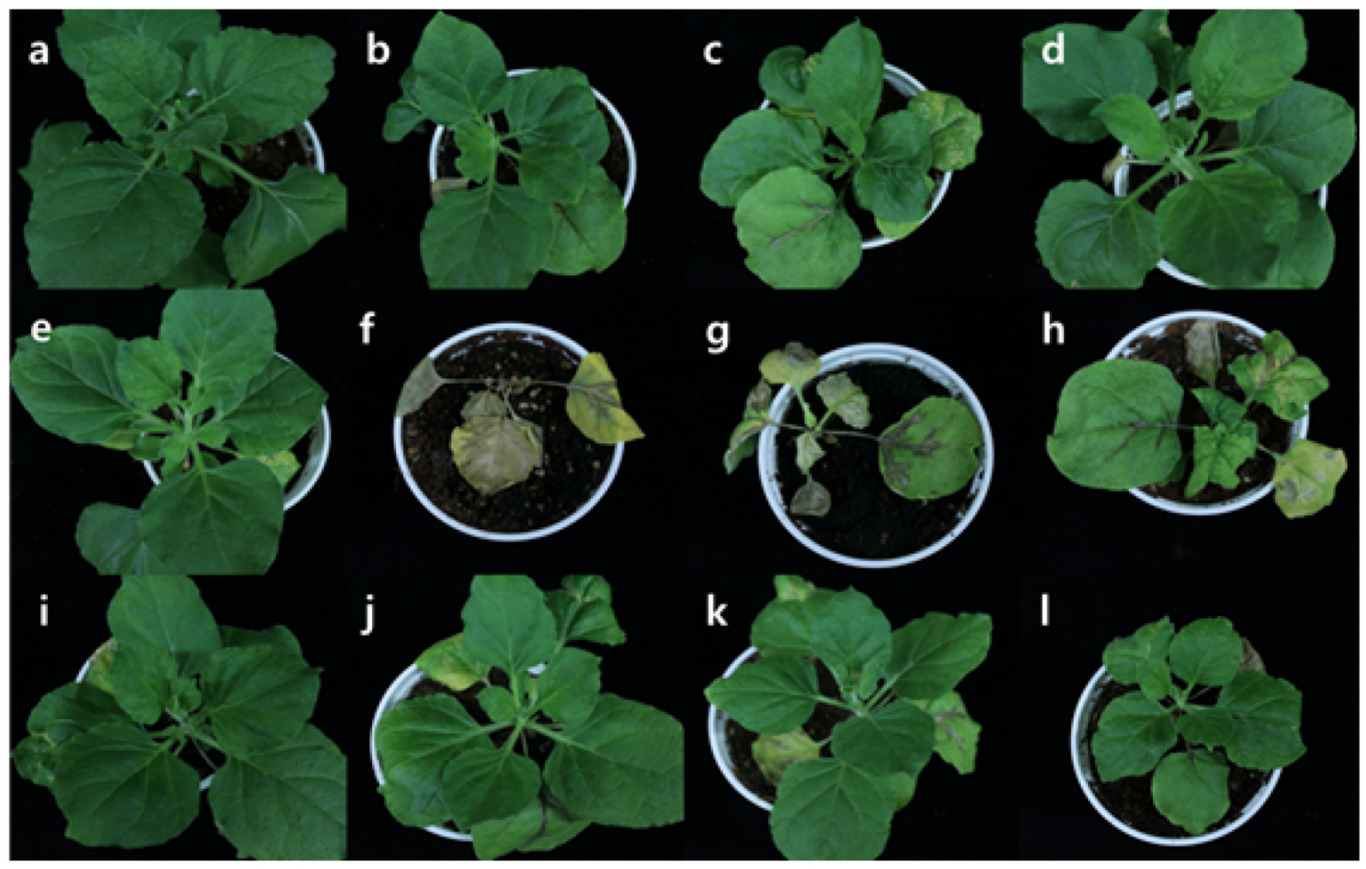
Developmental phenotypes of *N*. *benthamiana* plants silenced for genes encoding putative host factors. Phenotypes of wild-type *N*. *benthamiana* (a) and VIGS lines for *Cysk* (b), *FDH* (c), *CRT3* (d), *AGP-S2* (e), *UBI11* (f), *ARF1* (g), *ARF* (h), *H3* (i), *ARD* (j), and *PPM* (k) at 12 days after TRV agro-infiltration. (l) TRV::00 was used as a negative control (empty vector).

To test whether the selected genes were required for CMV infection and plant development, the 10 candidate genes were silenced using a TRV-based VIGS system. VIGS was performed with 2 weeks old *N*. *benthamiana* plants using previously described protocols and silenced plants were compared with wild-type *N*. *benthamiana*. At 12 dpi, VIGS plants showed various developmental phenotypes, such as curved leaves, stunting, and arrested growth, depending on the targeted gene (Fig 2b–2k). Plants infected with TRV::00 (empty vector) were used as negative control (Fig 2l) and *Phytoene desaturase* (PDS)-silenced plants were used as a silencing control (data not shown). Among 10 candidate genes, VIGS plants for *UBI11*, *ARF1*, and *ARF* showed severe developmental defects at 6 dpi and yellow leaves at 10 dpi (data not shown). Eventually, the VIGS plants of these three lines died at 12 dpi (Fig 2f–2h). TRV::00 plants showed typical TRV symptoms such as curved leaves and slow growth compared to wild type. The VIGS lines for *Cysk*, *FDH*, *CRT3*, *AGPase*, *H3*, *ARD*, and *PPM* showed similar phenotypes to the TRV::00 control plants (Fig 2). These results demonstrate that VIGS targeting of *Cysk*, *FDH*, *CRT3*, *AGPase*, *H3*, and *ARD* genes does not affect plant growth and that these lines are suitable for studying CMV infectivity in *N*. *benthamiana*.

**Fig 3:**
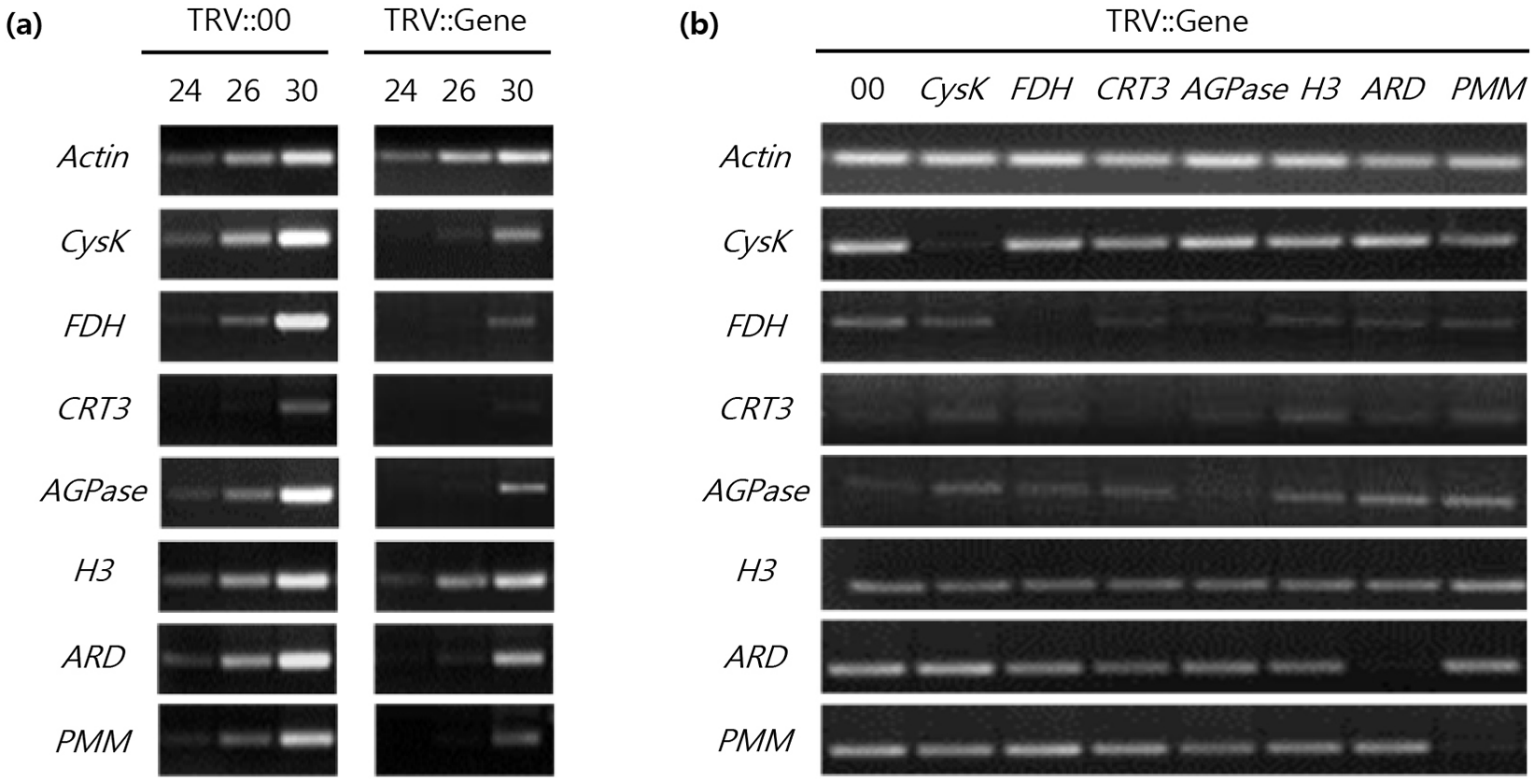
Relative expression of targeted host factor in silenced plants. **(a)** The expression level of each candidate gene was evaluated by semi-quantitative RT-PCR analysis at 12 d after TRV agro-infiltration. *N*. *benthamiana Actin* transcript was used as a standard. Each candidate gene cDNA was amplified by PCR with 24, 26, and 30 cycles. TRV::00 was used for comparison with candidate gene-silenced plants. **(b)** Examination of co-silencing of *Cysk*, *FDH*, *CRT3*, *AGPase*, *H3*, *ARD*, and *PMM* genes by semi-quantitative RT-PCR analysis at 12 dpi in each individual VIGS line.

To check expression levels of the targeted genes, semi-quantitative RT-PCR (S2 Table) was performed using gene-specific primers. Infiltrated TRV::00 (empty vector) plants were used a positive control. The mRNA expression levels of *Cysk*, *FDH*, *CRT3*, *AGPase*, and *ARD* were significantly reduced in VIGS lines at 12 dpi compared to TRV::00 plants. However, *H3* expression was not reduced in H3 VIGS plants (Fig 3a). We also checked for co-silencing of candidate genes in VIGS plants for each gene, and found no effects of the expression of other candidate genes (Fig 3b).

### Effects on CMV-P1 Infection of Silencing of the Candidate Genes

**Fig 4:**
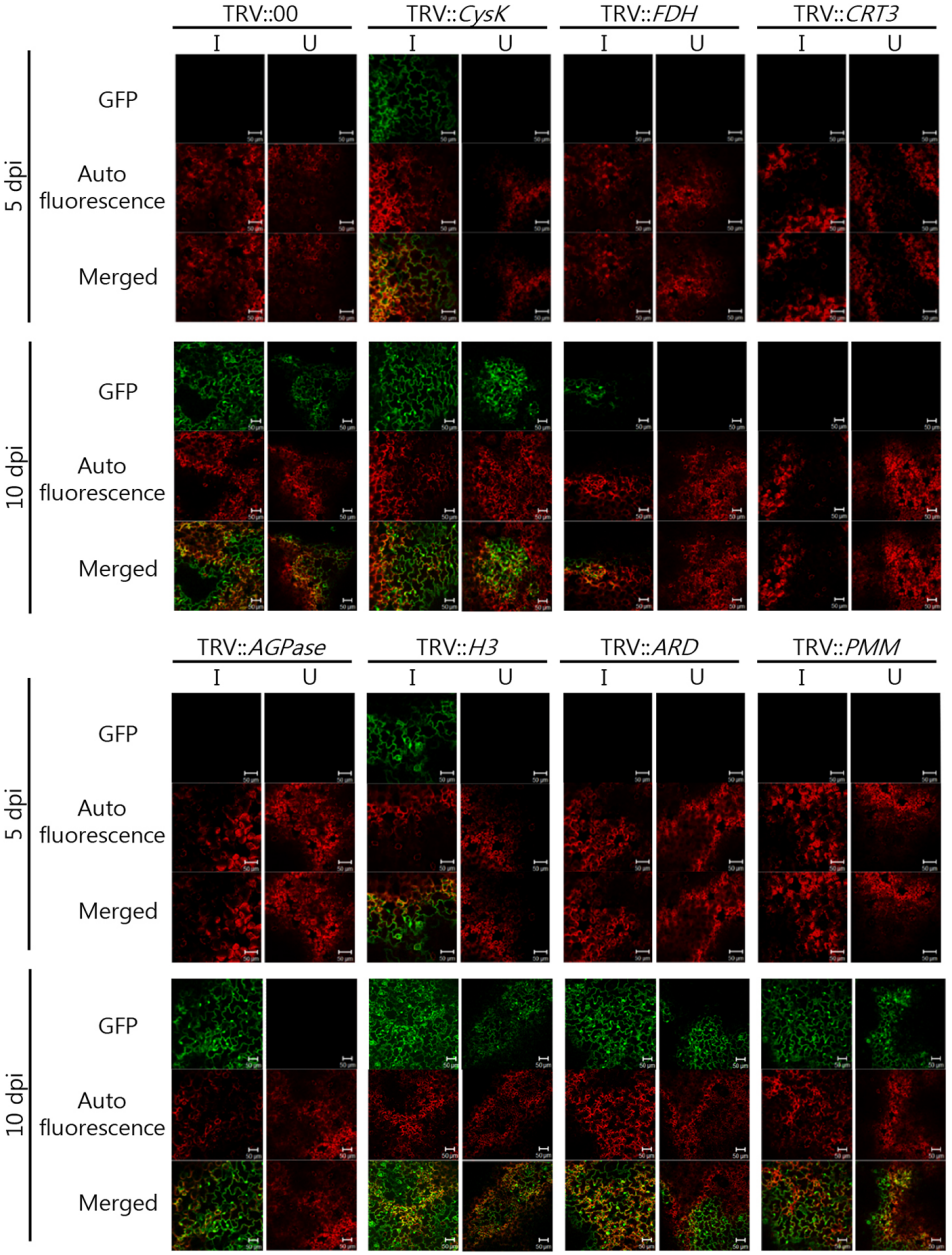
Effects of gene silencing on CMV-P1-GFP infection in *N*. *benthamiana*. GFP fluorescence was observed at 5 dpi and 10 dpi in VIGS plants, and TRV::00 plants were used as a positive control. Images on the left are optical sections of the inoculated leaves, and those on the right are optical sections in the upper leaves. Images top to bottom are GFP, autofluorescence, and merged images, respectively. The green fluorescence signal indicates CMV-P1 expressing GFP, and red fluorescence signal indicates chloroplasts. Scale bars = 50 μm.

To investigate the effects of silencing each candidate gene on CMV-P1 infection, CMV-P1-GFP was inoculated into the upper two leaves of VIGS plants. We monitored GFP florescence of the CMV-P1 virus using a confocal laser-scanning microscope at 5 dpi and 10 dpi (Fig 4). After CMV-P1 infection, the inoculated leaves of the TRV::00 control line showed strong GFP fluorescence at 10 dpi, although no fluorescence was detected at 5 dpi. Systemic infection was also detected in the un-inoculated upper leaves at 10 dpi. The *CysK* VIGS plants showed GFP signals in the inoculated leaves at both 5 dpi indicating that they are more susceptible to CMV infection. In the inoculated leaves of *AGPase*-silenced plants, GFP fluorescence was detected strongly at 10 dpi although no fluorescence was detected in the un-inoculated upper leaves. We also detected GFP signal in the both inoculated and uninoculated leaves of *ARD* and *PMM*-silenced plants at 10 dpi. These results indicate that *Cysk*, *AGPase*, *H3*, *ARD*, and *PMM* are not main factors for amplification of CMV-P1. The *FDH*-silenced plants showed weak GFP signal in inoculated leaves, but no GFP signal in uninoculated leaves. In the case of *CRT3*-silenced plants, there was no CMV-P1-GFP signal in the either the inoculated or the uninoculated, upper leaves at 5 dpi and 10 dpi.

To confirm the virus infection in the candidate gene-silenced plants, CMV-P1 coat protein (CP) accumulation was detected by ELISA at 5 and 10 dpi using leaf discs of the inoculated and uninoculated upper leaves in each line. ELISA analysis showed the same trend as the GFP-based analysis (S1 Fig). CP accumulation was not detected in inoculated or un-inoculated leaves of *FDH*-, *CTR3*-, *ARD*-, and *PMM*-silenced plants at 5 dpi, whereas a reduction of CP accumulation at 10 dpi was observed only in *FDH*- and *CTR3*-silenced plants (S1 Fig).

**Fig 5:**
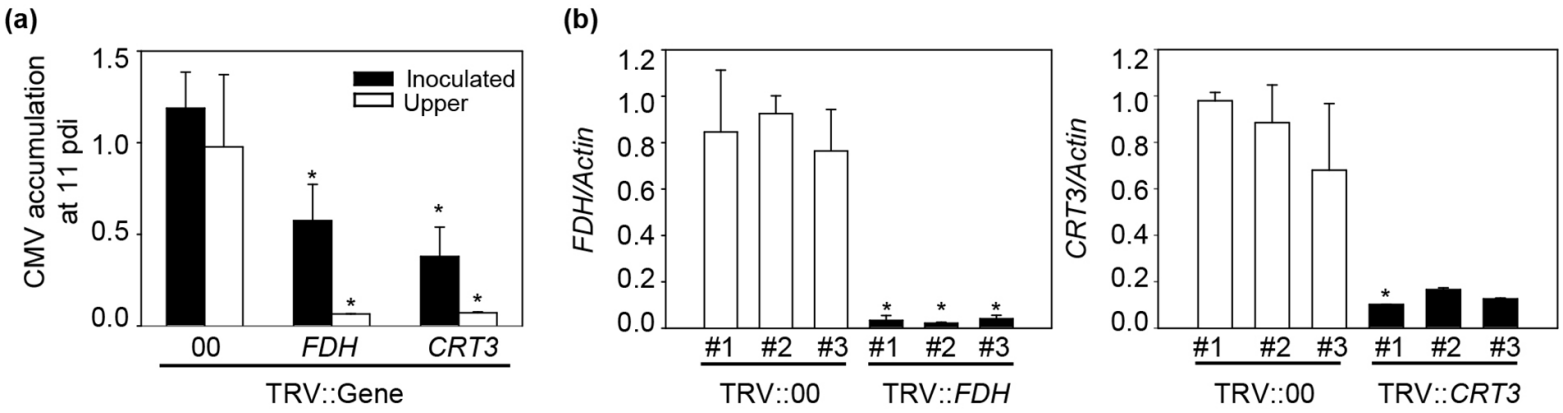
CMV accumulation in *FDH*- and *CRT3*-silenced *N*. *benthamiana*. (**a**) CMV accumulation in plants silenced for *FDH* and *CRT3* genes. Virus accumulation was detected by ELISA. Two leaf discs of the inoculated and upper leaves of the inoculated plants were sampled at 11 dpi. Means and standard deviations were obtained from three biological replicates. (**b**) The mRNA expression levels of *FDH* and *CRT3* were evaluated by real-time PCR analysis at 11 dpi. To normalize the expression level, *N*. *benthamiana Actin* transcript was used. Two leaf discs of the inoculated leaves were sampled at 11 dpi. Empty vector was the positive control. #1, #2, and #3 represent biological replicates. Asterisks indicate statistically significant differences relative to the empty vector as determined by Student’s *t*-test (*P< 0.05).

To confirm that *FDH* and *CRT3* are necessary for CMV infection, more detailed analysis was performed. Again, CMV-P1-GFP was inoculated into *N*. *benthamiana* plants after silencing of *FDH* and *CRT3* genes. After infection, the inoculated leaves of TRV::00 showed strong GFP fluorescence at 11 dpi although weak fluorescence was detected in the uninoculated leaves. However, we did not detect GFP signal at 11 dpi in either inoculated or uninoculated leaves of *FDH*- and *CRT3*-silenced plants (S2 Fig). CMV accumulation was also significantly decreased in the *FDH*- and *CRT*-silenced plants (Fig 5a). To confirm that the expression levels of the targeted genes were reduced at that time point, real-time PCR was performed using gene-specific primers (S2 Table). Infiltrated TRV::00 (empty vector) plants were used a positive control. The mRNA expression levels of *FDH* and *CRT3* were significantly reduced at 11 dpi in the corresponding VIGS lines compared with TRV::00 plants (Fig 5b). Taken together, these results suggest that *FDH* and *CRT3* are an essential factors for CMV-P1 infection in plants.

## Discussion

In this study, we identified novel host factors that interacted with the CMV-P1 helicase domain for CMV infection. We found that *N*. *benthamiana* plants silenced for the *FDH* or *CRT3* gene showed decreased infection by CMV-P1, indicating that *FDH* and *CRT3* are required for CMV-P1 infection in the plant.

FDH is one of the most abundant soluble proteins in mitochondria and is found in various organisms such as bacteria, yeast, and plants. FDH catalyzes the oxidation of formate (HCOO-) into CO_2_ [28, 41]. FDH has a putative mitochondrial signal peptide for targeting to mitochondria [42], although Arabidopsis *FDH* is localized in both mitochondria and chloroplasts [43, 44]. Mitochondria are involved in programmed cell death and the hypersensitive response [45]. In plants, FDH has been reported to function in various stress responses. FDH transcriptional and translational accumulation are induced by stresses such as hypoxia, chilling, drought, dark, wounding, and iron deficiency [46]. In addition, *FDH* is involved in biotic stress responses. *FDH* is upregulated by the fungus *Colletotrichum lindemuthianum* in *Phaseolus vulgaris* and by *Phytophthora citricola* in *Fagus sylvatica* [28, 47]. In *N*. *benthamiana*, *Sonchus yellow net virus* and *Impatiens necrotic spot virus* affect *FDH* expression [38]. The mitochondrial *FDH* is up-regulated by both *Sonchus Yellow net virus* (SYNV) and *Impatiens necrotic spot virus* (INSV) infection in *N*. *benthamiana* [38]. In *F*. *sylyatica* seedlings, *FDH* is induced by infection with the root pathogen *P*. *citricola* [47]. These reports indicate that *FDH* plays a significant role in pathogenicity in plants. In pepper plants silenced for *FDH1*, bacteria grew rapidly and expression of defense-related genes such as *PR1*, *PR10*, and *DEF1* was decreased, indicating that *FDH1* functions in bacterial disease defense [29]. Indeed, pepper *FDH1* transcriptional and translational expression is increased by *Xanthomonas campestris* pv. *vesicatoria*. Here, we showed that *FDH*-silencing in *N*. *benthamiana* inhibited infection by CMV-P1 virus (Fig 4, S1 Fig, and Fig 5). Furthermore, we found that pepper FDH directly interacted with helicase domain of CMV-P1 in yeast (Fig 1). These results suggest that FDH has a role in the pathogenesis of viral pathogen as well as bacterial and fungal pathogens.

CRT is a calcium-binding protein in the endoplasmic reticulum (ER) lumen with an established role as a molecular chaperone [48, 49]. CRT has been reported to play crucial roles in plants including in reproduction [50, 51], tissue regeneration [52, 53], abiotic stress responses [54, 55], and immunity [31, 56, 57]. Phylogenetic studies and expression analysis revealed that higher plants contain two distinct groups of CRTs: a CRT1/CRT2 group and a CRT3 group [58]. *Arabidopsis* CRT1 complements the chaperone functions and calcium storage capacity of mouse CRT, and functions as an alleviator of endoplasmic reticulum (ER) stress [59]. Recently, it was reported CRT2 functions through its N-terminal domain as a self-modulator that can possibly prevent the salicylic acid-mediated runaway defense responses triggered by its C-terminal calcium-buffering activity in response to pathogen invasion [57]. Furthermore, CRT3 is needed for the accumulation of bacterial elongation factor Tu receptor (EFR), a pattern-recognition receptor that is responsible for pathogen-associated molecular pattern-triggered immunity. These findings suggested a role for CRT3 in regulation of plant defense against pathogens [56].

We showed that CRT3 protein directly interacts with helicase domain of CMV-P1 (Fig 1), indicating that CRT3 mediates CMV-P1 in virus infection. In *N*. *tabacum*, tobacco mosaic virus movement protein (TMV MP) interacts with CRT for cell-to-cell transport [31]. In *Arabidopsis*, *CRT* genes are involved in virus defense pathways. In Arabidopsis, *CRT* expression is induced after inoculation with *Turnip vein clearing virus* (TVCV), *Oilseed rape mosaic virus* (ORMV), *Potato virus X* (PVX), CMV strain Y, or TuMV [30]. Here, we found that CMV-P1 infection requires *CRT3* (Fig 4). These results suggest that *CRT* genes might be common factors in various virus infection pathways.

In conclusion, we demonstrated that FDH and CRT3 proteins physically interact with the helicase domain of CMV-P1 (Fig 1) and that FDH and CRT3 function in CMV-P1 infection (Figs 4 and 5). These results suggest that *FDH* and *CRT3* mutations or knockouts may provide a new strategy for breeding CMV resistance in crop plants.

## Supporting Information

S1 Fig CMV accumulation in plants silenced for candidate host factors. Virus accumulation was detected by ELISA. Two leaf discs of the inoculated and upper leaves of the inoculated plants were sampled at 5 dpi and 10 dpi. PC and NC indicate positive and negative control, respectively. Asterisks indicates significant differences relative to the empty vector control as determined by Student’s *t*-test (*P < 0.*05*).
(TIF)

S2 Fig Effects of gene silencing on CMV-P1-GFP infection in *N*. *benthamiana*. (TIF)

S1 Table Primers used in this study. (DOCX)

S2 Table Host genes identified by yeast two-hybrid analysis. (DOCX)
