# Supplementary figures and images for: Isolation and Characterization of Pepper Genes Interacting with the CMV-P1 Helicase Domain
Source: PLoS One. 2016 Jan 11;11(1):e0146320. doi: 10.1371/journal.pone.0146320 (PMC4709182; doi:10.1371/journal.pone.0146320)

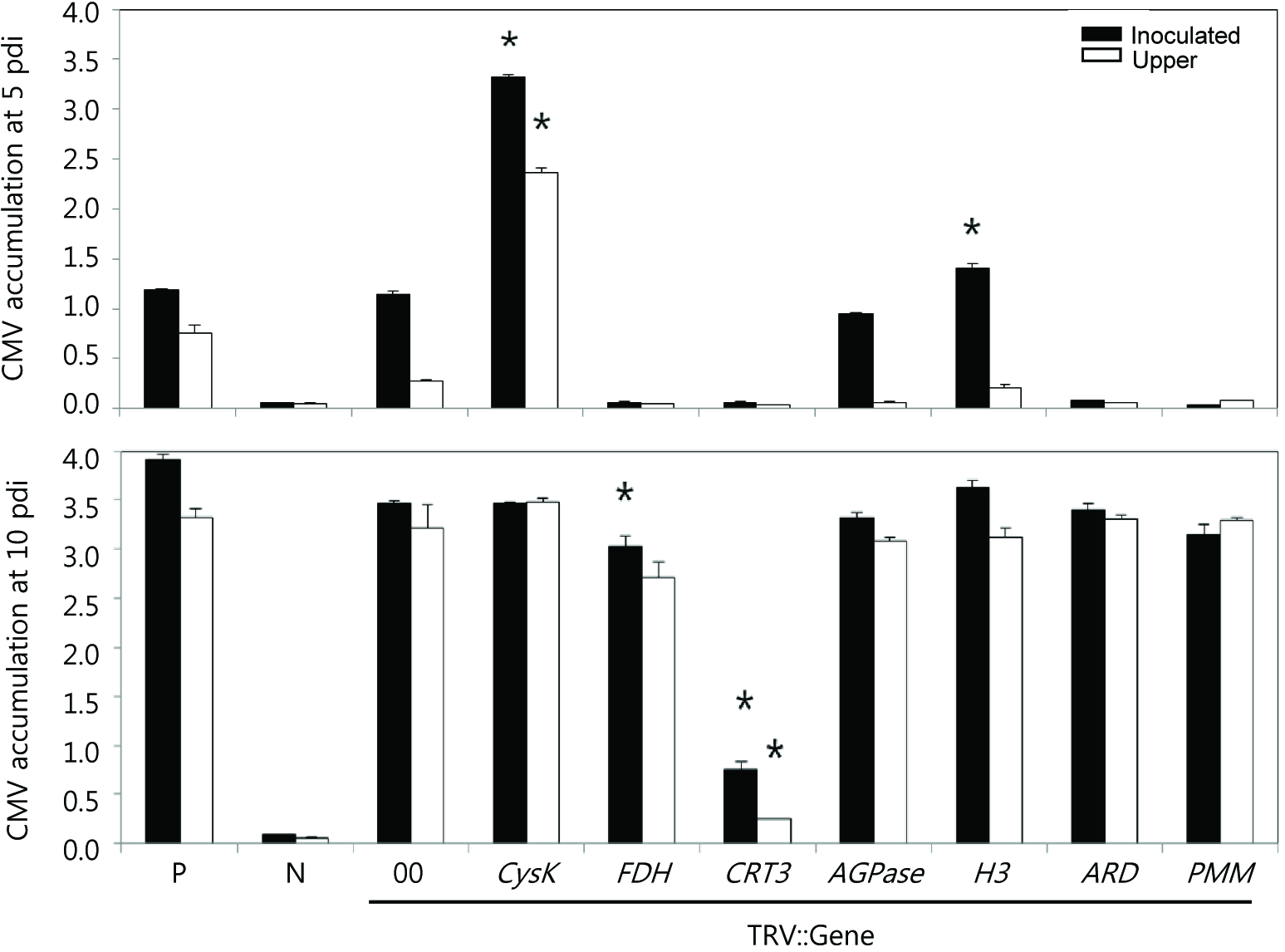

Supplement: S1 Fig — Virus accumulation was detected by ELISA. Two leaf discs of the inoculated and upper leaves of the inoculated plants were sampled at 5 dpi and 10 dpi. PC and NC indicate positive and negative control, respectively. Asterisks indicates significant differences relative to the empty vector control as determined by Student’s t-test (*P < 0.05). (TIF) [file pone.0146320.s001.tif]

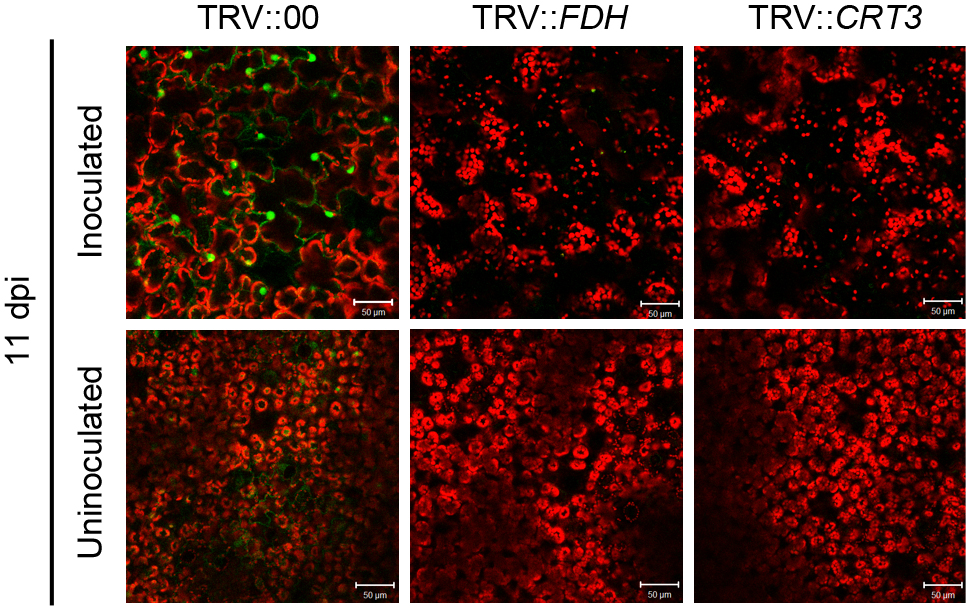

Supplement: S2 Fig — (TIF) [file pone.0146320.s002.tif]
