# Supplementary material for: Isolation and Characterization of Pepper Genes Interacting with the CMV-P1 Helicase Domain
Source: PLoS One. 2016 Jan 11;11(1):e0146320. doi: 10.1371/journal.pone.0146320 (PMC4709182; doi:10.1371/journal.pone.0146320)
Supplement: S1 Table — (DOCX) [file pone.0146320.s003.docx]

**S1 Table. List of primers used in this study**

| **Name** | **Sequence (5’ → 3’)** | **Application** |
| --- | --- | --- |
| ADMCS_F | CCCCACCAAACCCAAAAAAAGAG | PCR screening |
| ADMCS_R | GTTTTTCAGTATCTACGATTCATAGATCT | PCR screening |
| CysK_LIC_F | CGACGACAAGACCCTTCAACAATCCCTTCACTTC | TRV-LIC (VIGS) |
| CysK_LIC_R | GAGGAGAAGAGCCCTACTGCAACATGGCTCC | TRV-LIC (VIGS) |
| FDH_LIC_F | CGACGACAAGACCCTCTGGGACTACCATTGATG | TRV-LIC (VIGS) |
| FDH_LIC_R | GAGGAGAAGAGCCCTAGCGGACATGTTTCCA | TRV-LIC (VIGS) |
| CRT3_LIC_F | CGACGACAAGACCCTTATCCGATCAAAAAGGATC | TRV-LIC (VIGS) |
| CRT3_LIC_R | GAGGAGAAGAGCCCTTCATCCCAGTGATCAGG | TRV-LIC (VIGS) |
| ADP_LIC_F | CGACGACAAGACCCTGTCATTTGAAGATTCAATCTT | TRV-LIC (VIGS) |
| ADP_LIC_R | GAGGAGAAGAGCCCTTTGCAGCTAAAACCTCC | TRV-LIC (VIGS) |
| UBI11_LIC_F | CGACGACAAGACCCTCGGCTGAGAGGTGGTA | TRV-LIC (VIGS) |
| UBI11_LIC_R | GAGGAGAAGAGCCCTTCTGAACTCTCAACCTCCA | TRV-LIC (VIGS) |
| ARF1_LIC_F | CGACGACAAGACCCTCTTTCCCTAACCTTTTTCA | TRV-LIC (VIGS) |
| ARF1_LIC_R | GAGGAGAAGAGCCCTAGACCCTGAGTGTTCTGG | TRV-LIC (VIGS) |
| ARF_LIC_F | CGACGACAAGACCCTGGAGGACGAGAACGAGAA | TRV-LIC (VIGS) |
| ARF_LIC_R | GAGGAGAAGAGCCCTTCTCCACAAAGGACGAATC | TRV-LIC (VIGS) |
| H3_LIC_F | CGACGACAAGACCCTCTTGTTCGTGAAATTGCT | TRV-LIC (VIGS) |
| H3_LIC_R | GAGGAGAAGAGCCCTTCCACAACCATTGATACCT | TRV-LIC (VIGS) |
| ARD_LIC_F | CGACGACAAGACCCTAGGAAGTGGTTATTTTGAT | TRV-LIC (VIGS) |
| ARD_LIC_R | GAGGAGAAGAGCCCTACAAAGATTTCAAGTTCTTC | TRV-LIC (VIGS) |
| PPM_LIC_F | CGACGACAAGACCCTAAAGAGAGGAACAT‍TCGTT | TRV-LIC (VIGS) |
| PPM_LIC_R | GAGGAGAAGAGCCCTGTCATTTCCTCCCTTGTAT | TRV-LIC (VIGS) |
| TRV-LIC insert-F | TGTTACTCAAGGAAGCACGATGAGCT | Sequencing |
| TRV-LIC insert-R | CAGGCACGGATCTACTTAAAGAACGTAG | Sequencing |
| Nb-actin-F | CCAGGTATTGCTGATAGAATGAG | RT-PCR |
| Nb-actin-R | CTGAGGGAAGCCAAGATAGAG | RT-PCR |
| Nb_CysK_F | AAGGACAGGATAGGGTTCAGTATG | RT-PCR |
| Nb _CysK _R | CAAATTGTTGAAGGATATAGGCATC | RT-PCR |
| Nb _FDH_F | AAAGCAAACGAATATGCTGAAAT | RT-PCR |
| Nb _FDH_R | CCAGTGACCTCTGCTACTGTCAA | RT-PCR |
| Nb _CRT3_F | TTCTTTGAAGAAAGATTTGATGATG | RT-PCR |
| Nb _CRT3_R | ATAACATGAAGTTTCTTCGTCTGTGTA | RT-PCR |
| Nb _ADP_F | CCCAGACTCCAGGGGATG | RT-PCR |
| Nb _ADP_R | GGTTTCTCAGCAAATTGGATGAT | RT-PCR |
| Nb_H3_F | CATAAAGTTCCTCTTCTCCTTCTGTAGT | RT-PCR |
| Nb_H3_R | ACGAAGAGCAACGGTTCCA | RT-PCR |
| Nb_ARD_F | GAGGACGTCGTACAGGCATG | RT-PCR |
| Nb_ARD_R | GCAACACAGTAACGGATCTCTTC | RT-PCR |
| Nb_PPM_F | CATGAGATCTACGAGTCCGAGAG | RT-PCR |
| Nb_PPM_R | AAACAAAACGTACTACATTCATATTATAATTTT | RT-PCR |

Adaptor sequences for LIC (Ligation Independent Cloning) are underlined.
