# Supplementary material for: Isolation and Characterization of Pepper Genes Interacting with the CMV-P1 Helicase Domain
Source: PLoS One. 2016 Jan 11;11(1):e0146320. doi: 10.1371/journal.pone.0146320 (PMC4709182; doi:10.1371/journal.pone.0146320)
Supplement: S2 Table — (DOCX) [file pone.0146320.s004.docx]

**S2 Table. List of host genes identified by a yeast two-hybrid analysis**

| **Clone number** | **Description** | **Species of**  **functional study** | **Copy number** | **Relation with**  **pathogenesis** |
| --- | --- | --- | --- | --- |
| 11-2-21 | Ethylene-responsive element binding protein | *Capsicum annuum* | 1 |  |
| 11-2-4178 | Phosphomannomutase | *Solanum lycopersicum* | 1 | Viral pathogen |
| 11-3-17 | Formate dehydrogenase | *Solanum lycopersicum* | 2 | Viral pathogen |
| 12-1-20 | Calreticulin-3 precursor | *Zea mays* | 1 | Viral pathogen |
| 12-1-32 | Antimicrobial peptide snakin | *Capsicum annuum* | 1 |  |
| 12-2-44 | Photosystem I subunit XI | *Nicotiana attenuata* | 1 |  |
| 12-2-59 | Histone H2B | *Capsicum annuum* | 1 |  |
| 12-3-76 | Hexameric polyubiquitin 6PU11 | *Capsicum annuum* | 1 | Viral pathogen |
| 12-4-77 | 24K germin like protein | *Nicotiana tabacum* | 1 |  |
| 12-5-4 | Chloroplast ferredoxin-NADP^+^ oxidoreductase | *Capsicum annuum* | 1 |  |
| 12-5-6 | Cysteine synthase | *Capsicum annuum* | 1 | Viral pathogen |
| 12-5-12 | Methylketone synthase Ib | *Solanum lycopersicum* | 1 |  |
| 12-6-37 | 40S ribosomal protein S12 | *Vitis vinifera* | 1 |  |
| 12-6-45 | Photosystem I reaction centre PSI-D subunit precursor | *Solanum tuberosum* | 1 |  |
| 12-6-49 | Thionin-like protein | *Nicotiana tabacum* | 1 |  |
| 12-6-53 | Ribosomal protein L3 | *Solanum lycopersicum* | 1 |  |
| 12-7-57 | Membrane channel protein | *Medicago sativa* | 1 |  |
| 12-7-64 | Translation factor SUI1 | *Brachypodium distachyon* | 1 |  |
| 12-7-68 | Secretory peroxidase | *Nicotiana tabacum* | 1 |  |
| 12-8-66 | ADP-glucose pyrophosphorylase | *Lycopersicon esculentum* | 1 | Viral pathogen |
| 12-8-83 | Polyketide cyclase/dehydrase and  lipid transport protein | *Arabidopsis thaliana* | 1 |  |
| 12-8-94 | ADP-ribosylation factor 1 | *Nicotiana benthamiana* | 1 | Fungal pathogen |
| 12-8-98 | Enolase | *Solanum lycopersicum* | 2 |  |
| 12-9-42 | Membrane steroid-binding protein | *Medicago truncatula* | 1 |  |
| 12-9-102 | Ribulose 1,5-bisphosphate carboxylase | *Capsicum annuum* | 1 |  |
| 12-9-123 | ADP-ribosylation factor | *Capsicum annuum* | 1 | Fungal pathogen |
| 12-10-106 | Peptide transporter PTR3-A | *Medicago truncatula* | 1 |  |
| 12-10-107 | Ribosomal protein L12 | *Nicotiana tabacum* | 1 |  |
| 12-10-108 | Glycine-rich RNA-binding protein | *Nicotiana sylvestris* | 1 |  |
| 12-11-114 | Glycoprotein-like protein | *Solanum tuberosum* | 1 |  |
| 12-11-116 | Photosystem II 23 kda protein | *Solanum lycopersicum* | 1 |  |
| 12-12-119 | Ribosomal protein L25 | *Solanum tuberosum* | 1 |  |
| 12-12-132 | Histone H3 | *Capsicum annuum* | 1 | Virus infection |
| 12-13-18 | 18S ribosomal protein | *Lycium barbarum* | 1 |  |
| 12-13-52 | TCP transcription factor 14 | *Solanum lycopersicum* | 1 |  |
| 12-13-144 | Acireductone dioxygenase | *Solanum tuberosum* | 1 | Fungal pathogen |
| 12-17-124 | 40S ribosomal protein S23 | *Ricinus communis* | 1 |  |
| 12-17-125 | NADP-dependent isocitrate dehydrogenase | *Nicotiana tabacum* | 1 |  |
